# Awareness, Preferences, and Willingness to Download Pregnancy Health Management Apps Among Women in Louisiana: Cross-Sectional Survey

**DOI:** 10.2196/92287

**Published:** 2026-09-16

**Authors:** Priyanka Chaudhary, Alexis Bourgeois, Renee Underwood, Melissa Martin, Kara Denstel, Leanne Redman, Amanda Staiano

**Affiliations:** 1 Pediatric Obesity and Health Behavior Laboratory Pennington Biomedical Research Center Baton Rouge, LA United States; 2 Pennington Biomedical Research Center Southeastern Louisiana University Hammond, LA United States; 3 Population and Public Health Sciences Pennington Biomedical Research Center Baton Rouge, LA United States; 4 Reproductive Endocrinology & Women's Health Pennington Biomedical Research Center Baton Rouge, LA United States

**Keywords:** maternal health, health apps, mobile health, mHealth, intention to use, user acceptance

## Abstract

**Background:**

Pregnancy is a critical life stage that requires comprehensive health management. Pregnancy health management apps offer accessible support for prenatal and postpartum care, yet behavioral factors and preferences influencing their adoption remain unclear.

**Objective:**

We aimed to describe confidence, awareness, perceived health priorities, preferred app features, and willingness to download or pay for pregnancy health management apps among women in Louisiana. The primary outcome was willingness to download a pregnancy health management app.

**Methods:**

A cross-sectional, web-based, 10-minute, structured survey was conducted by MESH LLC from March 2025 to April 2025 among 597 women in Louisiana via the Alchemer platform. Eligible respondents identified as women and either had children aged <21 years or had no children but were pregnant or hoped to become pregnant. The survey comprised six domains: (1) sociodemographic and pregnancy characteristics, (2) confidence in maintaining a healthy pregnancy weight, (3) awareness of pregnancy health apps, (4) perceived importance of pregnancy-related health topics, (5) preferred app features, and (6) willingness to download or pay for a pregnancy health management app. Items were multiple-choice, Likert-scale, or ranked. Data were analyzed using descriptive statistics, exact tests for bivariate comparisons, and ordinal logistic regression.

**Results:**

Respondents were predominantly aged 31 to 40 years (258/597, 43.2%), married (359/597, 60.1%), and college educated (205/597, 34.3%). Most respondents (321/597, 53.8%) reported being very or extremely confident in maintaining a healthy weight during pregnancy. Among respondents routed to the survey item assessing the likelihood of downloading a pregnancy health management app, 94.8% (201/212) were “somewhat likely,” “very likely,” or “definitely willing” to download a pregnancy health management app. The most frequently downloaded or used apps were Pregnancy & Baby Tracker (224/597, 37.5%) and BabyCenter (211/597, 35.3%). Managing stress and mental health (mean 8.36, SD 2.34), along with understanding which foods are safe during pregnancy (mean 8.21, SD 2.36), ranked as the most important health priorities. Among respondents routed to the preferred-feature and payment-preference questions, postpartum support (135/205, 65.9%), kick counters and contraction timers (113/205, 55.1%), and bump trackers and photo progress tools (112/205, 54.6%) were among the most desired app features. Adjusted analyses showed limited evidence of sociodemographic differences in willingness. Respondents with annual household incomes of >US $150,000 had lower odds of reporting greater willingness than those with incomes of <US $75,000 (adjusted odds ratio 0.36, 95% CI 0.15-0.87; *P*=.02); however, the overall multivariable model was not statistically significant (likelihood-ratio *χ*^2^_13_=17.8; *P*=.17).

**Conclusions:**

Women demonstrated high willingness to download pregnancy health management apps, with a strong interest in mental health, nutrition, and postpartum support. Willingness was high across the pregnancy-relevant subgroup, with limited evidence of consistent sociodemographic differences in download intention. Future studies should examine actual app uptake, engagement, and effectiveness in diverse pregnant and postpartum populations.

## Introduction

Pregnancy is a critical and transformative stage in a woman’s life, characterized by significant physiological, emotional, and behavioral changes that require comprehensive health management to optimize outcomes for both mother and child [1,2]. Monitoring nutrition, weight, stress, pre-existing medical conditions, and physical activity is important to support healthy gestation [3,4]. Effectively managing these factors helps maintain maternal well-being and reduce the risk of adverse outcomes such as gestational diabetes, hypertensive disorders, and perinatal complications [5-7]. The gestational period offers a unique opportunity to encourage positive health behaviors that can have long-term effects on both maternal and child health [8,9].

Pregnancy health management apps have become increasingly available as sources of health information and self-management support during pregnancy and the postpartum period [10,11]. It is estimated that 73% of the global population uses the internet, with the majority (96%) of users accessing it through mobile phones (ie, smartphones) [12]. The growth in smartphone ownership worldwide has encouraged the widespread adoption of health apps [13]. Apps specific to pregnancy provide women with educational content, appointment reminders, symptom trackers, diet and weight monitoring, and access to social support platforms [14-16]. More than half of pregnant women in middle-to-high income nations use a pregnancy-related app, indicating widespread acceptance of digital tools for pregnancy health management [17-19].

Previous studies have reported associations between pregnancy app use and maternal knowledge, self-management, and engagement with prenatal care [20-23]. For example, a systematic review suggests that digital health interventions, including pregnancy-related apps, may be associated with increased use of antenatal care services, improved weight management during pregnancy, and better maternal and perinatal outcomes [20]. However, user engagement, cultural relevance, evidence-based material, and app quality (eg, functionality, information accuracy, and user satisfaction) have a significant impact on the efficacy and long-term use of these apps [17,24]. Technical issues, lack of personalization, limited postpartum content, and privacy concerns have been consistently identified as barriers to long-term app use [25-27]. Moreover, digital health disparities related to socioeconomic status, education, and cultural beliefs influence awareness, access, and desire to use mobile health (mHealth) tools [28]. Despite the growing availability of pregnancy-related apps, limited information is available regarding awareness, preferences, and willingness to use these apps among diverse groups of women, particularly those living in underserved communities [29]. Understanding user preferences may help inform the development and dissemination of pregnancy health management apps that are responsive to the needs of diverse populations.

Louisiana is an important setting for this survey because it faces substantial maternal health challenges, including high maternal mortality rates, persistent racial disparities in pregnancy-related outcomes, and limited access to maternity care in many communities. Although pregnancy-related apps are increasingly available, disparities in awareness and use remain. To address this knowledge gap, a market research survey was conducted among women in Louisiana to assess awareness, perceptions, and preferences related to pregnancy health management apps. Therefore, the objectives of this survey were to (1) assess women’s confidence, awareness, and perceived importance of pregnancy-related health management apps; (2) identify preferred app features and evaluate willingness to download and pay for such apps; and (3) examine sociodemographic and pregnancy-related factors associated with willingness to download a pregnancy health management app. The primary outcome was willingness to download a pregnancy health management app.

## Methods

### Research Design

This was a cross-sectional, web-based market survey conducted via the Alchemer (Alchemer LLC) platform. The survey targeted women residing in Louisiana and was conducted from March 26 to April 2, 2025. This survey was part of a broader initiative aimed at informing the development of a culturally appropriate, evidence-based pregnancy health management app. The target sample size was approximately 600 respondents, as determined by the market research firm to provide stable estimates of consumer preferences across key demographic groups while remaining feasible within the available project resources. This survey followed the STROBE (Strengthening the Reporting of Observational Studies in Epidemiology) reporting guidelines for cross-sectional studies. A completed STROBE checklist is provided as Multimedia Appendix 1 [30].

### Participants and Sample Size

Respondents were recruited through an advertising agency (MESH LLC) using their in-house online panel, which includes more than 20,000 Louisiana residents. Respondents were considered eligible if they (1) identified as a woman and (2) had children aged <21 years or had no children but were pregnant or hoped to become pregnant in the near future. This broad eligibility was intentional, reflecting a market research design aimed at capturing perspectives from the full range of potential app users, including women who anticipated a future pregnancy rather than limiting recruitment to those currently pregnant. Recruitment emails containing a unique survey link were distributed to eligible panel members. Interested respondents reviewed an electronic information page describing the purpose of the survey and voluntary participation before proceeding with the survey. The survey took approximately 10 minutes to complete and included multiple-choice and Likert-scale items.

### Survey Instrument

#### Overview

Respondents completed a structured survey consisting of six modules assessing (1) sociodemographic and pregnancy characteristics, (2) confidence in managing a healthy weight, (3) awareness and use of pregnancy health apps, (4) perceived importance of pregnancy health management factors, (5) preferred app features, and (6) willingness to download and pay for a pregnancy health app. The instrument was developed by a professional market research firm (MESH LLC) based on prior mHealth, pregnancy, and consumer health app literature.

#### Demographic Information

The survey captured sociodemographic characteristics (age group, marital status, education, and household income), family composition (number of children aged <21 years), and pregnancy status (recently given birth, currently pregnant, hoping to become pregnant in the near future, or no recent pregnancy).

#### Confidence in Pregnancy Weight Management

A single-item question was used to assess respondents’ confidence in maintaining a healthy weight during pregnancy. Respondents rated their confidence using a 5-point Likert scale ranging from 1=“not at all confident” to 5=“extremely confident,” with an additional option for those with 0=no experience.

#### Pregnancy-Related App Awareness and Use

Respondents were asked about their awareness and use of 10 popular pregnancy-related apps, including Amila (Amila Tech Ltd), BabyCenter (Everyday Health Group), and Glow Nurture Pregnancy (Glow Inc). For each app, respondents selected one of the following three response options: (1) unaware of this app, (2) familiar with the app but have not downloaded it, or (3) have downloaded or used it. This approach allowed the evaluation of both brand recognition (awareness) and active engagement (use) across several pregnancy health management apps.

#### Importance of Health Management Factors

Respondents were asked to rate the importance of 11 pregnancy-related health topics for maintaining a healthy lifestyle during pregnancy. Each topic was scored on a 10-point scale (1=“not at all important” to 10=“extremely important”). These topics included managing pregnancy and postpartum weight, ensuring proper nutrition for the baby, understanding food safety, dealing with pregnancy cravings and emotional eating, finding safe and effective exercise, managing morning sickness and fatigue, balancing pre-existing medical conditions, managing stress and mental health, accessing maternity health services, and receiving support from family and society. Mean (SD) scores were calculated for each factor and ranked in descending order based on mean importance values, with higher scores indicating greater perceived importance among respondents.

#### Preferred App Features

The survey asked respondents to rate 10 possible app features on a 3-point scale: 3=“definitely needed,” 2=“nice to have,” or 1=“not necessary.” These features consisted of weight tracking, calorie counting, recipes and food suggestions, articles and guides, videos, community support and sharing, baby size comparison, bump tracking and photo progress, postpartum support, and kick counting and contraction timing.

#### Willingness to Download and Pay for a Pregnancy Health Management App

The survey assessed willingness to download and pay for a pregnancy health management app. The first question evaluated respondents’ willingness to download a pregnancy health management app using a 5-point scale ranging from 1=“would not” to 5=“definitely would,” along with a “not sure” response option.

The second question asked about preferred payment options, with response categories of 1=“free version only,” 2=“free with ads or limited features,” 3=“1-time fee for full access,” 4=“monthly or yearly subscription,” and 5=“free version but pay for extra features.” Respondents who indicated a willingness to pay were also asked, through an open-ended question, to specify the amount they would be willing to spend. For descriptive and bivariate analyses, willingness to download was dichotomized as willing (“somewhat likely,” “very likely,” or “definitely would”) vs not willing (“not very likely” or “would not”). For the primary multivariable analysis, the full 5-level ordinal response scale was retained. Responses of “not sure” were excluded from willingness analyses because they were outside the ordered likelihood scale.

### Survey Skip Logic and Participant Routing

The survey included programmed skip logic to route respondents to questions relevant to their pregnancy experience and responses to preceding items (Multimedia Appendix 2). All 597 respondents completed the sociodemographic, confidence, and pregnancy app awareness sections. The perceived importance of health management factors was administered to a pregnancy-relevant subgroup identified through the programmed routing logic, resulting in 27.6% (165/597) of respondents completing this section.

A total of 216 respondents were routed to the survey item assessing the likelihood of downloading a pregnancy health management app (Q5) through either the screening item assessing pregnancy status among respondents with children (S5) or the screening item assessing pregnancy status and future pregnancy intentions among respondents with no children (S6). Only 1.9% (4/216) of respondents selected “not sure,” leaving 98.1% (212/216) of respondents with classifiable willingness responses. Of these, 94.8% (201/212) selected “somewhat likely,” “very likely,” or “definitely would,” whereas 5.2% (11/212) selected “not very likely” or “would not.”

Per the programmed skip logic, the 5.2% (11/212) of respondents who selected “not very likely” or “would not” were routed directly to the survey item assessing confidence in the ability to maintain a healthy weight (Q9) and did not complete the pregnancy app feature or payment questions. The remaining 94.9% (205/216) of respondents, including the 1.9% (4/216) of respondents who selected “not sure,” continued to the preferred-feature and payment-preference questions. Consequently, analyses of classifiable willingness responses are based on a sample size of 212 respondents, whereas preferred-feature and payment-preference analyses are based on a sample size of 205 respondents. Respondents who did not meet the programmed criteria for the pregnancy-specific modules were routed past these questions. Accordingly, denominators differ across survey domains and should not be interpreted as missing responses from the full sample.

### Data Analysis

All analyses were conducted using SPSS (version 29.0; IBM Corp). Descriptive statistics were used to summarize respondents’ sociodemographic and pregnancy-related characteristics. Categorical variables were expressed as frequencies and percentages, and continuous variables were expressed as means and SDs.

For analyses of the primary outcome, respondents who selected “not sure” at Q5 (4/216, 1.9%) were excluded because this response was not part of the ordered willingness scale. This resulted in 212 respondents for descriptive analyses of willingness. Available-case denominators varied slightly for education and household income because of missing or “prefer not to answer” responses. For the adjusted ordinal logistic regression, cases with missing values for any included covariate were excluded using listwise deletion, resulting in an analytic sample of 207 respondents. Pregnancy status responses obtained through the survey were harmonized into recently given birth, currently pregnant, or hoping to become pregnant in the near future for analyses of the pregnancy-specific willingness outcome.

The primary outcome, willingness to download a pregnancy health management app, was measured on a 5-point ordinal scale (1=“would not” to 5=“definitely would”) for the primary multivariable analysis and dichotomized as willing (“somewhat likely,” “very likely,” or “definitely would”) vs not willing (“not very likely” or “would not”) for descriptive and bivariate analyses. Because only 5.2% (11/212) of respondents were classified as not willing, several contingency table cells contained small, expected counts. Therefore, exact tests were used to examine bivariate associations between willingness and age group, marital status, education, household income, number of children, and pregnancy status. The Fisher exact test was used for 2×2 comparisons, and Fisher-Freeman-Halton exact tests with Monte Carlo estimation were used for larger contingency tables. Cramér V was reported as an effect size measure. Original survey categories were retained for these descriptive bivariate comparisons.

For the primary multivariable analysis, willingness to download a pregnancy health management app was analyzed using an ordinal logistic regression (proportional odds) model that retained all 5 response categories. The adjusted model included age group, marital status, education level, household income, pregnancy status, and number of children aged <21 years as covariates. The original categories for each predictor were retained in the multivariable model. Regression coefficients, adjusted odds ratios (AORs), and 95% CIs were reported.

Prior to multivariable modeling, multicollinearity was assessed using variance inflation factors (VIFs; all VIFs <2.0). Overall model fit was evaluated using the likelihood-ratio chi-square test and pseudo-*R*^2^ statistics. Statistical significance was set at *P*=.05 (2-tailed).

### Ethical Considerations

A human research determination request was submitted to the Pennington Biomedical Research Center Institutional Review Board (IRB). The IRB reviewed the protocol and determined that the marketing survey was exempt from full board review under 45 CFR 46.101(b)(2), as it involved anonymous survey procedures in which responses were recorded in such a manner that participants could not be identified. Accordingly, no formal IRB application number was assigned. Before beginning the survey, all participants were presented with an electronic information page describing the survey purpose, voluntary nature of participation, and data collection procedures before proceeding. The information page explicitly stated that completion of the survey would be taken as implied permission to participate. No personally identifiable information was collected, and responses were analyzed anonymously. Respondents received a small monetary incentive (<US $1.00 per respondent) for survey completion through digital payment platforms (eg, PayPal).

## Results

### Respondent Characteristics

A total of 597 eligible respondents completed the survey. Respondents were predominantly aged 31 to 40 years, married, and had a college degree or higher or some college or technical school education. Most households reported earning <US $75,000 per year, and the largest proportion had 1 or 2 children in the household. Among 558 respondents with children who completed the pregnancy status question, the majority reported no recent pregnancy, while smaller proportions had recently given birth, were currently pregnant, or hoped to become pregnant in the near future. Detailed frequencies and percentages for all sociodemographic characteristics are presented in Table 1.

**Table 1:** Respondent characteristics (N=597).

Characteristics		Respondents, n (%)
**Age (years)**		
	≤30	162 (27.1)
	31-40	258 (43.2)
	41-50	133 (22.3)
	≥51	41 (6.9)
	Prefer not to respond	3 (0.5)
**Marital status**		
	Married	359 (60.1)
	Not married	236 (39.5)
	Prefer not to answer	2 (0.3)
**Education**		
	High school or less	184 (30.8)
	Some college or technical school	197 (33.0)
	College degree or higher	205 (34.3)
	Prefer not to answer	11 (1.8)
**Annual household income (US $)**		
	<75,000	335 (56.1)
	75,000-150,000	177 (29.6)
	>150,000	65 (10.9)
	Prefer not to respond	20 (3.4)
**Number of children in household**		
	0	39 (6.5)
	1	229 (38.4)
	2	207 (34.7)
	≥3	122 (20.4)
**Pregnancy status^a^**		
	Recently given birth	46 (8.2)
	Currently pregnant	40 (7.2)
	Hope to become pregnant in the near future	100 (17.9)
	No recent pregnancy	372 (66.7)

^a^Pregnancy status percentages are based on the 558 respondents with children who completed this item.

### Confidence in Maintaining a Healthy Pregnancy Weight

Respondents’ confidence in maintaining a healthy weight during pregnancy varied (N=597). Most reported being very confident (n=162, 27.1%) or extremely confident (n=159, 26.6%). Among those expressing lower confidence, 64 (10.7%) reported being not very confident, and 31 (5.2%) reported being not at all confident. A small proportion (n=20, 3.4%) of respondents had no prior experience with weight management during pregnancy.

### Awareness of Pregnancy Health Management Apps

Awareness of pregnancy health management apps varied considerably across respondents (N=597; Multimedia Appendix 3). The apps most frequently downloaded or used were Pregnancy & Baby Tracker (Everyday Health Inc; n=224, 37.5%), BabyCenter (n=211, 35.3%), and What to Expect (Ziff Davis Inc; n=193, 32.3%). However, a substantial proportion of respondents were unaware of several pregnancy-related apps, such as Amila (n=405, 67.8%) and Sprout Pregnancy (Med ART Studios; n=365, 61.1%).

### Perceived Importance of Health Management Factors

Among the 165 respondents who completed the importance rating module, all 11 health management factors received mean importance ratings of >7 on the 10-point scale (Table 2). Managing stress and mental health received the highest mean importance rating (mean 8.36, SD 2.34), followed by understanding safe foods during pregnancy (mean 8.21, SD 2.36) and managing weight while ensuring proper nutrition for the baby (mean 8.05, SD 2.36). Managing morning sickness and fatigue (mean 7.96, SD 2.35), balancing prenatal health with pre-existing conditions (mean 7.81, SD 2.49), and getting support from family and society (mean 7.68, SD 2.58) also received high ratings. The remaining topics also received relatively high ratings, with mean ratings ranging from 7.50 (SD 2.53) to 7.61 (SD 2.40).

**Table 2:** Mean importance rating of pregnancy-related health management factors (N=165).

Factors	Rating^a^, mean (SD)
Managing stress and mental health	8.36 (2.34)
Understanding what foods are safe or unsafe during pregnancy	8.21 (2.36)
Managing weight while ensuring proper nutrition for the baby	8.05 (2.36)
Managing morning sickness and fatigue	7.96 (2.35)
Balancing prenatal health with pre-existing medical conditions (eg, diabetes and hypertension)	7.81 (2.49)
Getting support from family and society for a healthy pregnancy lifestyle	7.68 (2.58)
Managing weight during pregnancy or postpartum	7.61 (2.40)
Preparing for postpartum weight management and recovery	7.55 (2.49)
Dealing with pregnancy cravings and emotional eating	7.53 (2.58)
Finding safe and effective exercises during pregnancy	7.52 (2.47)
Accessing maternity health care and support services for weight management	7.50 (2.53)

^a^Ratings were based on a 10-point scale (1=“not at all important” to 10=“extremely important”). Items are presented in descending order by mean importance.

### Preferred App Features

Among the 205 respondents who completed the pregnancy app features module, postpartum support was the most commonly rated “definitely needed” feature, followed by recipes and food suggestions, a kick counter and contraction timer, and a bump tracker and photo progress tool (Table 3). Weight tracking was also rated as definitely needed by approximately half of respondents. The remaining features, including baby size comparison, calorie counting, articles and guides, videos, and community support and sharing, were rated as either “definitely needed” or “nice to have” by most respondents.

**Table 3:** Preferred pregnancy health app features (N=205).

Features	Definitely needed, n (%)^a^	Nice to have, n (%)^a^	Not necessary, n (%)^a^
Postpartum support	135 (65.9)	57 (27.8)	13 (6.3)
Recipes and food suggestions	120 (58.5)	64 (31.2)	21 (10.2)
Kick counter and contraction timer	113 (55.1)	72 (35.1)	20 (9.8)
Bump tracker and photo progress	112 (54.6)	76 (37.1)	17 (8.3)
Weight tracking	103 (50.2)	87 (42.4)	15 (7.3)
Baby size comparison	98 (47.8)	86 (42.0)	21 (10.2)
Community support and sharing	90 (43.9)	92 (44.9)	23 (11.2)
Videos	90 (43.9)	87 (42.4)	28 (13.7)
Articles and guides	89 (43.4)	86 (42.0)	30 (14.6)
Calorie counting	80 (39.0)	95 (46.3)	30 (14.6)

^a^Percentages may not total 100.0% because of rounding.

### Willingness to Download and Pay for an App

Among the 212 respondents with classifiable willingness responses, 201 (94.8%) indicated willingness to download the app: 107 (50.5%) reported that they “definitely would,” 63 (29.7%) were “very likely,” and 31 (14.6%) were “somewhat likely.” In contrast, 7 (3.3%) were “not very likely,” and 4 (1.9%) reported that they “would not” download the app.

Among the 205 respondents who completed the payment-preference questions, the largest proportion indicated a preference for a 1-time fee for full access (n=57, 27.8%), followed by a preference for a free version only (n=43, 21.0%), a monthly or yearly subscription (n=42, 20.5%), a free version with optional paid features (n=35, 17.1%), and a free version with advertisements or limited features (n=28, 13.7%).

### Sociodemographic and Pregnancy-Related Factors Associated With Willingness to Download a Pregnancy Health Management App

Exact bivariate analyses were performed to examine associations between respondents’ sociodemographic and pregnancy-related factors and their willingness to download a pregnancy health management app (Table 4). Number of children was associated with willingness to download the app (exact *P*=.01; Cramér V=0.206). Age group (exact *P*=.58), marital status (*P*>.99), education (exact *P*=.63), household income (exact *P*=.34), and pregnancy status (exact *P*=.76) were not statistically associated with willingness. Given the small number of respondents categorized as not willing (n=11), these bivariate findings should be interpreted cautiously.

**Table 4:** Bivariate analysis of willingness to download a pregnancy health management app (N=212)^a^.

Variables			Not willing, n (%)		Willing to download, n (%)		Exact *P* value^b^		Cramér V^c^
**Age group (years; n=212)**							.58		0.084
	≤30	7 (63.6)		93 (46.3)					
	31-40	3 (27.3)		88 (43.8)					
	41-50	1 (9.1)		17 (8.5)					
	≥51	0 (0.0)		3 (1.5)					
**Marital status (n=212)**							>.99		0.009
	Married	7 (63.6)		124 (61.7)					
	Not married	4 (36.4)		77 (38.3)					
**Education (n=210)**							.63		0.068
	High school or less	2 (18.2)		58 (29.1)					
	Some college or technical school	5 (45.5)		65 (32.7)					
	College degree or higher	4 (36.4)		76 (38.2)					
**Household income (US $; n=208)**							.34		0.112
	<75,000	4 (36.4)		102 (51.8)					
	75,000-150,000	3 (27.3)		61 (31.0)					
	>150,000	4 (36.4)		34 (17.2)					
**Number of children in household (n=212)**							*.01* ^d^		0.206
	0	4 (36.4)		25 (12.4)					
	1	0 (0.0)		75 (37.3)					
	2	5 (45.5)		66 (32.8)					
	≥3	2 (18.2)		35 (17.4)					
**Pregnancy status (n=212)**							.76		0.059
	Recently given birth	3 (27.3)		43 (21.4)					
	Currently pregnant	1 (9.1)		38 (18.9)					
	Hope to become pregnant in the near future	7 (63.6)		120 (59.7)					

^a^Respondents who selected “not sure” for willingness to download (n=4) were excluded. Willingness was defined as “somewhat likely,” “very likely,” or “definitely would”; not willing was defined as “not very likely” or “would not.”

^b^Fisher exact test was used for the 2×2 marital status comparison; Fisher-Freeman-Halton exact tests with Monte Carlo estimation were used for multicategory comparisons.

^c^Cramér V is reported as an effect size measure.

^d^Italicized *P* value indicates statistical significance at *P*<.05.

The adjusted ordinal logistic regression included 207 respondents with complete data (Table 5). The overall model was not statistically significant (likelihood-ratio *χ*^2^_13_=17.8; *P*=.17). Most sociodemographic and pregnancy-related characteristics were not independently associated with willingness to download the pregnancy health management app. Compared with respondents with annual household incomes of <US $75,000, those reporting household incomes of >US $150,000 had lower odds of endorsing a higher willingness category (AOR 0.36, 95% CI 0.15-0.87; *P*=.02). Respondents hoping to become pregnant in the near future had approximately twice the odds of endorsing a higher willingness category than those who had recently given birth, although this association did not reach statistical significance (AOR 2.05, 95% CI 0.93-4.54; *P*=.08). Age, marital status, education, current pregnancy status, and number of children were not significantly associated with willingness. Given the nonsignificant overall model and limited variability in the outcome, individual coefficient estimates should be interpreted cautiously.

**Table 5:** Ordinal logistic regression model of willingness to download a pregnancy health management app (N=207)^a^.

Predictors			B^b^ (SE)		Wald *χ*^2^ (df)		*P* value		Adjusted odds ratio (95% CI)
**Age group** **(years; reference: ≤30 years)**									
	31-40	−0.311 (0.315)		1.0 (1)		.32		0.73 (0.39-1.36)	
	41-50	0.577 (0.559)		1.1 (1)		.30		1.78 (0.60-5.33)	
	≥51	−1.207 (1.143)		1.1 (1)		.29		0.30 (0.03-2.81)	
Marital status: not married (reference: married)			0.298 (0.348)		0.7 (1)		.39		1.35 (0.68-2.67)
**Education** **(reference: high school or less)**									
	Some college or technical school	−0.122 (0.388)		0.1 (1)		.75		0.89 (0.41-1.89)	
	College degree or higher	0.289 (0.468)		0.4 (1)		.54		1.34 (0.53-3.34)	
**Household income** **(US $; reference: <75,000)**									
	75,000-150,000	−0.442 (0.373)		1.4 (1)		.24		0.64 (0.31-1.34)	
	>150,000	−1.024 (0.453)		5.1 (1)		.02		0.36 (0.15-0.87)	
**Pregnancy status (reference: recently given birth)**									
	Currently pregnant	−0.326 (0.442)		0.5 (1)		.46		0.72 (0.30-1.72)	
	Hope to become pregnant in the near future	0.718 (0.406)		3.1 (1)		.08		2.05 (0.93-4.54)	
**Number of children aged <21 years (reference: 0 children)**									
	1	0.749 (0.490)		2.3 (1)		.13		2.12 (0.81-5.53)	
	2	0.802 (0.502)		2.6 (1)		.11		2.23 (0.83-5.96)	
	≥3	0.925 (0.560)		2.7 (1)		.10		2.52 (0.84-7.56)	

^a^The outcome was the 5-level ordinal likelihood of downloading a pregnancy health management app (1=“would not” to 5=“definitely would”). “Not sure” responses were excluded. Adjusted odds ratio >1 indicates greater odds of endorsing a higher download-likelihood category. Log likelihood=−234.88; Akaike information criterion=503.76; Bayesian information criterion=560.42; McFadden pseudo-*R*^2^=0.036; Nagelkerke pseudo-*R*^2^=0.091; likelihood-ratio *χ*^2^_13_=17.8; *P*=.17

^b^B: ordinal logistic regression coefficient.

## Discussion

### Principal Findings

This cross-sectional survey examined awareness, preferences, and willingness to download and pay for pregnancy health management apps among women in Louisiana. Given the cross-sectional, descriptive design, findings should be interpreted as exploratory rather than confirmatory, and conclusions are intended to generate hypotheses for future research rather than to establish causal relationships. The findings indicate substantial interest in digital tools for pregnancy health management, aligning well with the rapid global increase in smartphone access and mHealth adoption. As smartphone ownership and app use continue to expand globally, pregnancy-related apps have become an increasingly common source of health information during pregnancy and the postpartum period [12]. These findings add to the evolving understanding that user-centered design and culturally tailored dissemination strategies may enhance the reach and effectiveness of pregnancy apps, especially in underresourced or underserved populations [31]. Our findings are consistent with recent evidence from Australia, where 94% of pregnant or recently pregnant women reported using pregnancy apps, primarily to track fetal development and maternal changes [32].

Our findings are consistent with the view that pregnancy is widely perceived as a critical period requiring comprehensive health management across multiple domains, including stress, nutrition, weight management, postpartum health, and social support [1,33-36]. Managing stress and mental health emerged as the highest-rated health priority, indicating the importance of psychosocial well-being as an integral component of maternal health [37,38] during both pregnancy and the postpartum period [39,40]. This aligns with prior studies emphasizing mental health challenges during pregnancy and postpartum and supporting the inclusion of mental health support features in pregnancy apps [7,18,20,40].

There was considerable variation in awareness and use of existing pregnancy apps, with some popular apps, such as Pregnancy & Baby Tracker and BabyCenter, having high overall awareness, while others were less known. These data suggest an uneven landscape of digital engagement. This finding is consistent with previous research showing that, despite widespread smartphone use, actual engagement and app downloads vary widely due to barriers, including app quality, accuracy of content, personalization, trust, and cultural relevance [10,15,17,26,29]. Therefore, addressing these barriers may be important for promoting equitable access and sustained app use, which in turn may be associated with improved maternal health outcomes.

Importantly, preferences for app features among women indicated a strong demand for postpartum support, kick counters and contraction timers, bump trackers, and weight tracking, reflecting respondents’ desire for comprehensive and practical tools throughout pregnancy and after birth. This finding is consistent with prior qualitative studies, suggesting that users value apps that offer interactive, personalized, and targeted features [14,16]. Among respondents with classifiable pregnancy app willingness responses, 94.8% indicated that they were at least “somewhat likely” to download the app. Willingness to pay among respondents who completed the payment-preference question was more varied, indicating that high interest in downloading an app does not necessarily translate into uniform willingness to pay for access. This highlights the importance of considering financial barriers and sustainable models incorporating cost-sharing or tiered access, consistent with previous studies [41-43].

The pregnancy-specific analyses provided limited evidence that willingness differed systematically across sociodemographic or pregnancy-related groups. Although the number of children was associated with willingness in the bivariate analyses, none of the individual number-of-children categories was significantly associated with willingness in the adjusted model. Respondents with annual household incomes of >US $150,000 had lower adjusted odds of reporting greater willingness than respondents with incomes of <US $75,000; however, the overall multivariable model was not statistically significant. The income association should therefore be interpreted cautiously, particularly given the limited variability in willingness and the exploratory nature of the analysis.

The limited number of significant associations may partly reflect the high overall willingness observed in the pregnancy-relevant subgroup. With 94.8% of respondents expressing at least some willingness to download the app, there was relatively little variation in the outcome available to distinguish demographic groups. This high level of receptivity is consistent with previous studies demonstrating widespread use and interest in pregnancy-related mobile apps among pregnant women [15,19,32]. Rather than suggesting that pregnancy app interest is concentrated within specific demographic subgroups, our findings indicate broadly high receptivity among respondents in the pregnancy-specific subgroup. Previous research also suggests that adoption and continued use of pregnancy apps may depend on factors beyond sociodemographic characteristics, including usability, perceived usefulness, information quality, personalization, and other aspects of the user experience [26,27]. Additional research with larger samples and greater variability in app adoption intentions is needed to identify factors that facilitate or hinder actual uptake.

This survey provides regional data from Louisiana, a population that faces distinct maternal health challenges, including a high burden of adverse pregnancy outcomes and persistent health disparities [9]. By exploring user preferences and app-related perceptions, these findings may help inform the design and dissemination of culturally relevant, evidence-based digital health interventions to support healthy pregnancies and postpartum health in Louisiana and similar settings. Future research should examine actual app adoption and engagement behaviors and evaluate whether preferred features are associated with sustained use and improved maternal health outcomes.

### Strengths and Limitations

This market research survey demonstrates several notable strengths. First, it included a large statewide sample of women across Louisiana to assess pregnancy app awareness and related health perceptions, along with pregnancy-relevant analytic subsamples for assessing pregnancy-specific priorities, preferred app features, and willingness to download and pay for an app. The inclusion of respondents across varied age groups, educational levels, income categories, and reproductive histories provided perspectives from a demographically and reproductively diverse sample. Second, by examining both awareness and willingness to pay, the study extends beyond general attitudes to offer actionable insights for developers, policymakers, and public health practitioners aiming to design, price, and promote effective pregnancy-related mobile apps. Third, the analysis examining the associations between willingness to download a pregnancy health management app and key sociodemographic characteristics contributes context to the growing body of literature on digital health equity and disparities in maternal care access.

Nevertheless, several limitations should be acknowledged. The cross-sectional design precludes causal inferences or assessment of changes in awareness and app interest over time. Because data were obtained through a convenience sample recruited from an online panel, respondents may have been more digitally literate or health-engaged than the general population, potentially leading to an overestimation of app awareness, confidence, and interest. Self-reported responses may also be subject to recall and social desirability bias. Second, although the overall survey included 597 respondents, pregnancy-specific analyses were based on smaller analytic subsamples.

The willingness analysis was based on 212 respondents with classifiable responses, and feature and payment analyses were based on 205 respondents. These findings therefore characterize the pregnancy-specific subgroup rather than the entire statewide sample and should not be generalized to all 597 respondents. Future studies should recruit larger samples of currently pregnant women and women planning pregnancy to confirm these findings. Third, willingness to download demonstrated a pronounced ceiling effect: 201 of 212 (94.8%) respondents with classifiable responses indicated at least some willingness, leaving only 11 (5.2%) respondents in the not-willing category. This limited variability reduced statistical power to detect sociodemographic differences and resulted in sparse cells in several bivariate comparisons. Exact tests were therefore used for bivariate analyses. Accordingly, the bivariate and multivariable association analyses should be considered exploratory. Fourth, age and income categories were predefined by the market research firm, and the broad groupings may have limited the ability to detect more nuanced sociodemographic differences. Finally, the narrow range of mean importance scores (7.50-8.36) may reflect a ceiling effect, limiting the discriminative capacity of the 10-point importance rating scale.

### Conclusions

This cross-sectional survey found substantial interest in pregnancy health management apps among women in Louisiana, particularly in features related to mental health, nutrition, postpartum support, and pregnancy tracking. Among respondents who completed the pregnancy-specific willingness question, willingness to download the app was high, with limited evidence of consistent sociodemographic differences in download intention. These findings may help inform user-centered development and dissemination of pregnancy health management apps. Future research should examine actual app adoption, sustained engagement, and health outcomes in larger and more diverse samples of pregnant women and women planning pregnancy.

## Appendix

Multimedia Appendix 1 STROBE checklist.

Multimedia Appendix 2 Survey instrument assessing pregnancy experiences, health management priorities, app awareness and preferences, and willingness to download and pay for an app.

Multimedia Appendix 3 Awareness and use of pregnancy health management apps among respondents.

## Abbreviations

AOR: adjusted odds ratio
IRB: Institutional Review Board
mHealth: mobile health
STROBE: Strengthening the Reporting of Observational Studies in Epidemiology
VIF: variance inflation factor

## Data Availability

The datasets obtained during the current survey are available from the corresponding author upon reasonable request.
