# Supplementary material for: Awareness, Preferences, and Willingness to Download Pregnancy Health Management Apps Among Women in Louisiana: Cross-Sectional Survey
Source: JMIR Form Res. 2026 Sep 16;10:e92287. doi: 10.2196/92287 (PMC13628032; doi:10.2196/92287)
Supplement: Supplementary file 2 [file formative_v10i1e92287_app2.docx]

**Greaux Healthy AP**

**General Market Survey Research- 2025**

**PROJECT SCOPE**

**Methodology: Web-based survey**

**Sample: 500 completed Interviews with Women with children or pregnant**

**S1  	Do you currently live in the state of Louisiana?**

 1= Yes	2 = No **[Thank & Terminate]**

**S2	Gender**

 1= Female	2 = Male **[Thank & Terminate]**

**S3**	**How many children do you have under 21 years of age?**

0 = None **[SKIP TO S6]**

_____ number of children

**S4	Do you have...  [CHECK ALL THAT APPLY]**

1 = Children not yet in school

2 = Children in elementary school (K through 5th grade)

3 = Children in middle school (6th through 8th grade)

4 = Children in high school (9th through 12th grade)

5 = Children in college

**S5**	**Which best reflects you?**

1 = Recently given birth

2 = Currently pregnant

3 = Hope to be pregnant near future

4 = No recent pregnancy

**ALL SKIP TO S7**

**S6	Which best reflects you?**

1 = Currently pregnant

2 = Hoping to be pregnant in the near future

3 = Hope to have a child in the next three years

4 = No current plans for children **[Thank & Terminate]**

**S7  	Your age**

1 = 30 or younger	3 = 41 to 50 years of age

2 = 31 to 40 years	4 = 50 and over

0=Prefer not to answer

Pregnancy Management App Questions

**1	How confident are/were you in maintaining a healthy weight during your pregnancy?**

5 = Extremely confident	2 = Not very confident

4 = Very confident	1 = Not at all confident

3 = Somewhat confident	0 = No experience

X = Prefer not to say

**2     	Please tell me if you are aware of any of these pregnancy management apps.**

| **ROTATE BRANDS** | Yes, downloaded this app | Familiar with the app but did NOT download | Unaware of the app |
| --- | --- | --- | --- |
| A. Amila | 3 | 2 | 1 |
| B. BabyCenter | 3 | 2 | 1 |
| C. Glow Nurture Pregnancy | 3 | 2 | 1 |
| D. Ovia Pregnancy Tracker | 3 | 2 | 1 |
| E. Pregnancy & Baby Tracker | 3 | 2 | 1 |
| F. Pregnancy Tracker Day by Day | 3 | 2 | 1 |
| G. Pregnancy tracker | 3 | 2 | 1 |
| H. Sprout Pregnancy | 3 | 2 | 1 |
| I. The Bump Pregnancy Tracker | 3 | 2 | 1 |
| J. What to Expect | 3 | 2 | 1 |

**IF Q1 EQ 2 AND S4 EQUALS 1,2,3 – Advance to Q3**

**IF Q2 EQ 2 AND S5 EQUALS 1 OR 2 – Advance to Q3**

**OTHERWISE,** **SKIP TO Q9**

**3	Managing a healthy lifestyle during pregnancy can be a challenge.  On a scale of one to ten, with 10 being extremely important and 1 being not at all important, how important are/were the following during your pregnancy?**

| **ROTATE ITEMS** | **Scale of Importance** |
| --- | --- |
| A Managing weight during pregnancy or postpartum |  |
| B Preparing for postpartum weight management and recovery |  |
| C Managing weight while ensuring proper nutrition for the baby |  |
| D Dealing with pregnancy cravings and emotional eating |  |
| E Finding safe and effective exercises during pregnancy |  |
| F Managing morning sickness and fatigue |  |
| G Understanding what foods are safe or unsafe during pregnancy |  |
| H Balancing prenatal health with pre-existing medical conditions (e.g., diabetes, hypertension) |  |
| I Managing stress and mental health |  |
| J Accessing maternity healthcare and support services for weight management |  |
| K Getting support from family and society for a healthy pregnancy lifestyle |  |

**4	Which two pregnancy health management factors are most important?**

First Mention:       ___________________

Second Mention:  ___________________

**5	If a health management pregnancy app was able to provide these features, how likely would you be to consider downloading the app?**

5 = Definitely would	2 = Not very likely **[SKIP TO 9]**

4 = Very likely	1 = Would NOT **[SKIP TO 9]**

3 = Somewhat likely	X = Not sure

**6	For each of the following, please tell me if this feature is definitely needed, would be nice to have, or not necessary for a health management pregnancy app.**

| **ROTATE ITEMS** | **Definitely**  **Needed** | **Nice to**  **Have** | **Not**  **Necessary** |
| --- | --- | --- | --- |
| A. Weight tracking | 3 | 2 | 1 |
| B. Calorie counting | 3 | 2 | 1 |
| C. Recipes and food suggestions | 3 | 2 | 1 |
| D. Articles and guides | 3 | 2 | 1 |
| E. Videos | 3 | 2 | 1 |
| F. Community support and sharing | 3 | 2 | 1 |
| G. Baby Size Comparison | 3 | 2 | 1 |
| H. Bump Tracker & Photo Progress | 3 | 2 | 1 |
| I. Postpartum Support | 3 | 2 | 1 |
| J. Kick Counter & Contraction Timer Count | 3 | 2 | 1 |

**7	Would you be willing to pay for such a health management pregnancy app?**

1 = No, I’d only use a free version. **[SKIP TO 9]**

2 = No, I'd only use a free version with ads or limited features. **[SKIP TO 9]**

3 = Yes, I’d pay a one-time fee for full access.

4 = Yes, I'd prefer a monthly or yearly subscription.

5 = Yes, I’d use a free version but pay for extra features.

**8	How much would you be willing to pay?**

_____ dollars

Personal Health Management App Questions

**9	How confident are you in your ability to maintain a healthy weight?**

5 = Extremely confident	2 = Not very confident

4 = Very confident	1 = Not at all confident

3 = Somewhat confident	X = Prefer not to say

**10     	Please tell me if you are aware of any of these health management apps.**

| **ROTATE BRANDS** | Yes, downloaded this app | Familiar with the app but did NOT download | Unaware of the app |
| --- | --- | --- | --- |
| A. MyFitnessPal | 3 | 2 | 1 |
| B. Noom | 3 | 2 | 1 |
| C. Lose It! | 3 | 2 | 1 |
| D. WW (formerly Weight Watchers) | 3 | 2 | 1 |
| E. Cronometer | 3 | 2 | 1 |
| F. Fitbit | 3 | 2 | 1 |
| G. HealthyOut | 3 | 2 | 1 |
| H. Fooducate | 3 | 2 | 1 |
| I. Lifesum | 3 | 2 | 1 |
| J. Strava | 3 | 2 | 1 |

**11	On a scale of one to ten, with 10 being extremely important and 1 being not at all important, how important are the following aspects of a health management app?**

| **ROTATE ITEMS** | **Scale of Importance** |
| --- | --- |
| A Ease of use & accessibility |  |
| B Personalized tracking & insights |  |
| C Goal setting & motivation |  |
| D Comprehensive health data integrated |  |
| E Educational resources & guidance |  |
| F Community & social features |  |
| G Evidence-based methodologies & resources |  |

**12	Health management apps provide you with the ability to develop healthy habits; help with weight management and design exercise programs for its users.  How likely would you be to consider downloading a personal health management app in the future?**

5 = definitely would	2 = not very likely

4 = very likely	1 = would NOT

3 = somewhat likely	X = Not sure

FAMILY Health Management App Questions

**14	How confident do you feel about helping your child/children maintain a healthy weight?**

5 = Extremely confident	2 = Not very confident

4 = Very confident	1 = Not at all confident

3 = Somewhat confident	X = Prefer not to say

**15	How often does the family eat home-cooked meals?**

1 = Daily	3 = 1 to 3 times a week

2 = 4 to 6 times a week	4 = Rarely / Never

X= Unsure

1**6	How would you describe your child/children's current weight?**

1 = underweight	3 = slightly overweight

2 = healthy weight	4 = overweight (but working to improve)

X= Unsure

**17	How often do your child/children engage in physical activity (e.g. sports, exercising, playing outside)?**

1 = Daily	3 = 1 to 3 times a week

2 = 4 to 6 times a week	4 = Rarely / Never

X= Unsure

**18	On a typical WEEKDAY, how many hours per day does your child use or watch a screen outside of school and homework (TV, phone, tablet, computer, video game)?**

0 = None	3 = Between 2 and 3 hours

1 = less than 1 hour	4 = Between 3 and 4 hours

2 = Between 1 to 2 hours	5 = More than 4 hours

X= Unsure

**19	On a typical WEEKEND, how many hours per day does your child use or watch a screen outside of school and homework (TV, phone, tablet, computer, video game)?**

0 = None	3 = Between 2 and 3 hours

1 = less than 1 hour	4 = Between 3 and 4 hours

2 = Between 1 to 2 hours	5 = More than 4 hours

X= Unsure

**20	Managing a healthy lifestyle for you and your family can be a challenge. On a scale of one to ten, with 10 being a serious concern and 1 being not at all a concern, how concerned are you with the following?**

| **ROTATE ITEMS** |  |
| --- | --- |
| A. Picky eating habits of children |  |
| B. Lack of time to prepare meals |  |
| C. Limited access to affordable healthy food |  |
| D. Child’s resistance to physical activities |  |
| E. High amount of daily screen time |  |
| F. Negative peer or family influence |  |
| G. Weight-related bullying, body image and self-esteem issues |  |
| H. Lack of understanding about nutritious food, balance, diet, portions etc. |  |
| I. Limited personal time for you to be able to focus on your growth & improvement |  |
| J. Finding reliable healthcare professionals & support programs focused on childhood weight issues |  |

**21	Which are your two biggest health concerns?**

[INCORPORATE ITEMS in Q21 plus Other specify: ________]

First Mention:       ___________________

Second Mention:  ___________________

**22	Family health management apps attempt to provide users with the tools necessary to develop long term healthy weight management habits.**

**Our goal is to build the most efficient and beneficial family wellness app in the market with your assistance.  Therefore, for each of the following, please indicate if the feature is definitely needed, would be nice to have, or is not necessary.**

| **ROTATE ITEMS** | **Definitely**  **Needed** | **Nice to**  **Have** | **Not**  **Necessary** |
| --- | --- | --- | --- |
| A. Tips for encouraging children’s physical activity | 3 | 2 | 1 |
| B. Guidance from pediatricians or nutritionists | 3 | 2 | 1 |
| C. Fun family activities to promote health | 3 | 2 | 1 |
| D. Ability to easily track all nutritional aspects: food, liquids, calories, weight, BMI | 3 | 2 | 1 |
| E. Exercise and physical activity monitoring (daily step counter) | 3 | 2 | 1 |
| F. Meal planning & grocery list integration | 3 | 2 | 1 |

**23	For each of the following, please indicate if the feature is definitely needed, would be nice to have, or is not necessary.**

| **ROTATE ITEMS**  **EDUCATION** | **Definitely**  **Needed** | **Nice to**  **Have** | **Not**  **Necessary** |
| --- | --- | --- | --- |
| G. Easy, quick healthy recipes | 3 | 2 | 1 |
| H. Visual guide (chart-infographic) to help with portion size, balance diet etc, | 3 | 2 | 1 |
| I. Advice from other parents/caretakers | 3 | 2 | 1 |
| J. Interactive quizzes for learning healthy habits | 3 | 2 | 1 |
| K. Video tutorials for workouts, meal prep, and mindfulness exercises | 3 | 2 | 1 |
| L. Evidence-based articles on weight management, health & nutrition | 3 | 2 | 1 |
| M. Expert Q & A chatbot to address health-related questions | 3 | 2 | 1 |

**24	For each of the following, please indicate if the feature is definitely needed, would be nice to have, or is not necessary.**

| **ROTATE ITEMS**  **REMINDERS & NOTIFICATION** | **Definitely**  **Needed** | **Nice to**  **Have** | **Not**  **Necessary** |
| --- | --- | --- | --- |
| A. Reminders & notifications regarding meals, liquids, nutrition supplements, vitamins, etc | 3 | 2 | 1 |
| B. Reminders & notifications regarding physical activity, exercising (daily step counter) | 3 | 2 | 1 |
| C. Providing goal-setting weekly updates | 3 | 2 | 1 |
| D. Create social network or community-shared challenges with other app members | 3 | 2 | 1 |
| E. Establish challenges/setting milestones or goals | 3 | 2 | 1 |

**25	Of all the items just mentioned, which THREE features do you feel are most VALUABLE?**

First Mention: _____________________________

Second Mention: _____________________________

Third Mention: _____________________________

Greaux Healthy App Perception Questions

**Greaux Healthy is an evidence-based public service initiative by LSU’s Pennington Biomedical Research Center in partnership with the state of Louisiana focused on preventing and treating childhood obesity in Louisiana. The app will be able to provide the features discussed.**

**26	How likely would you be to download the Greaux Healthy APP?**

5 = Definitely would	2 = Not very likely **[SKIP TO 29]**

4 = Very likely	1 = Would NOT **[SKIP TO 29]**

3 = Somewhat likely	X = Not sure

**27	Would you be willing to pay for such a family health management app?**

1 = No, I’d only use a free version. **[SKIP TO 29]**

2 = No, I'd only use a free version with ads or limited features. **[SKIP TO 29]**

3 = Yes, I’d pay a one-time fee for full access.

4 = Yes, I'd prefer a monthly or yearly subscription.

5 = Yes, I’d use a free version but pay for extra features.

**28	How much would you be willing to pay?**

____ dollars

**29	Which social media platforms do you frequently use?**

**[CHECK ALL THAT APPLY]**

A. Facebook

B. Instagram

C. LinkedIn

D. Pinterest

E. Reddit

F. Snapchat

G. Tik Tok

H. X orTwitter

I. WhatsApp

J. You Tube

K. None of these

**30	On average, how much time do you spend on social media daily?**

0 = zero/none                                                          	6 = 76 to 90 minutes

        	1 = 1 to 15 minutes                                                   	7 = 91 to 120 minutes

        	2 = 16 to 30 minutes                                                 	8 = 2 to 3 hours

        	3 = 31 to 45 minutes                                                 	9 = 3 to 4 hours

        	4 = 46 to 60 minutes                                                 	10 = Over 4 hours

        	5 = 61 to 75 minutes

**SAMPLE CHARACTERISTICS**

**The last few questions are needed for crosstab purposes only.**

**31	Are you married?**

        	1 = Yes

        	2 = No	0 = Prefer not to answer

**32	What best describes your level of education?**

1 = High school graduate or less                    4 = college undergraduate degree

2 = vocational/business/technical school        5 = postgrad/professional degree

3 = some college / two-year degree                0 = Prefer not to answer

**33	Total annual household income?**

        	1 = Under $75,000

        	2 = $75,000 to $150,000

        	3 = Over $150,000	0 = Prefer not to answer

**34	In which Parish do you live?**
