# Supplementary material for: Awareness, Preferences, and Willingness to Download Pregnancy Health Management Apps Among Women in Louisiana: Cross-Sectional Survey
Source: JMIR Form Res. 2026 Sep 16;10:e92287. doi: 10.2196/92287 (PMC13628032; doi:10.2196/92287)
Supplement: Supplementary file 3 [file formative_v10i1e92287_app3.docx]

**Supplemental Table 2. Awareness of pregnancy health apps (N = 597)**

| **App** | **Yes, downloaded this app**  **N (%)** | **Familiar, but did not download the app**  **N (%)** | **Unaware of the app**  **N (%)** |
| --- | --- | --- | --- |
| Amila | 74 (12.4) | 118 (19.8) | 405 (67.8) |
| BabyCenter | 211 (35.3) | 154 (25.8) | 232 (38.9) |
| Glow Nurture Pregnancy | 111 (18.6) | 164 (27.5) | 322 (53.9) |
| Ovia Pregnancy Tracker | 118 (19.8) | 155 (26.0) | 324 (54.3) |
| Pregnancy & Baby Tracker | 224 (37.5) | 169 (28.3) | 204 (34.2) |
| Pregnancy Tracker Day by Day | 129 (21.6) | 183 (30.7) | 285 (47.7) |
| Pregnancy Tracker | 158 (26.5) | 200 (33.5) | 239 (40.0) |
| Sprout Pregnancy | 64 (10.7) | 168 (28.1) | 365 (61.1) |
| The Bump Pregnancy Tracker | 129 (21.6) | 186 (31.2) | 282 (47.2) |
| What to Expect | 193 (32.3) | 190 (31.8) | 214 (35.8) |
